# Acceptability of risk‐stratified population screening across cancer types: Qualitative interviews with the Australian public

**DOI:** 10.1111/hex.13267

**Published:** 2021-05-11

**Authors:** Kate Dunlop, Nicole M. Rankin, Amelia K. Smit, Zofia Salgado, Ainsley J. Newson, Louise Keogh, Anne E. Cust

**Affiliations:** ^1^ Daffodil Centre The University of Sydney, a joint venture with Cancer Council NSW Sydney NSW Australia; ^2^ Melanoma Institute Australia The University of Sydney Sydney NSW Australia; ^3^ Sydney School of Public Health, The Faculty of Medicine and Health The University of Sydney Sydney NSW Australia; ^4^ Sydney Health Ethics, Sydney School of Public Health, The Faculty of Medicine and Health The University of Sydney Sydney NSW Australia; ^5^ Melbourne School of Population and Global Health The University of Melbourne Melbourne VIC Australia

**Keywords:** acceptability, cancer, implementation, risk‐stratified, risk‐tailored, screening

## Abstract

**Background:**

There is mounting evidence of the benefit of risk‐stratified (risk‐tailored) cancer population screening, when compared to standard approaches. However, shifting towards this approach involves changes to practice that may give rise to implementation challenges.

**Objectives:**

To explore the public's potential acceptance of risk‐stratified screening across different cancer types, including reducing screening frequency if at low risk and the use of personal risk information, to inform implementation strategies.

**Method:**

Semi‐structured interviews were conducted with 40 public participants; half had received personal genomic risk information and half had not. Participants were prompted to consider different cancers. Data were analysed thematically as one dataset.

**Results:**

Themes included the following: (a) a sense of security; (b) tailored screening is common sense; (c) risk and the need to take action; (d) not every cancer is the same; and (e) trust and belief in health messages. Both groups expressed similar views. Participants were broadly supportive of risk‐stratified screening across different cancer types, with strong support for increased screening frequency for high‐risk groups. They were less supportive of reduced screening frequency or no screening for low‐risk groups. Findings suggest the public will be amenable to reducing screening when the test is invasive and uncomfortable; be less opposed to forgo screening if offered the opportunity to screen at some stage; and view visible cancers such as melanoma differently.

**Conclusions:**

Approaching distinct cancer types differently, tailoring messages for different audiences and understanding reasons for participating in screening may assist with designing future implementation strategies for risk‐stratified cancer screening.

## 1 BACKGROUND

In comparison with the mostly ‘one size fits all’ approaches used in existing population screening for cancer, there is mounting evidence that risk‐stratified (risk‐tailored) screening can both improve the early detection of cancer for those most likely to benefit and reduce the well‐recognized harms of screening for those at lower risk.^1^, ^2^, ^3^, ^4^ Stratification of individuals into risk groups may be based on assessment of factors such as personal genomic and/or lifestyle risk information, as well as traditional risk factors such as age, family history or ethnicity. Shifting towards a risk‐stratified screening approach will involve changes to current practice, including recommending more frequent screening for those at high‐risk, different modalities of screening, and modified start and stop times. These changes may present challenges for implementation, for example the use of personal risk information to inform screening advice and a move towards less screening or potentially no screening for those at low risk.

To add to the evidence from modelling studies, several large trials are more definitively assessing the relative benefits and harms of risk‐stratified screening.^4^, ^5^, ^6^, ^7^, ^8^, ^9^ Successful implementation of tailored screening approaches will depend on it being accepted by end‐users, such as the general public.^10^, ^11^, ^12^ Acceptability in this setting refers to the perception that the use of additional risk factors and tailored screening advice is agreeable ^13^ and in the target population's interest^11^; therefore, increasing the likelihood that individuals will adhere to recommendations and benefit from improved clinical outcomes.^14^ Understanding the public's perceptions in the pre‐implementation stage of risk stratification will help inform strategies for successful implementation.^4^, ^15^, ^16^

Most existing research about the acceptability of risk‐stratified screening is in breast cancer,^11^, ^12^, ^17^, ^18^, ^19^, ^20^ but very little is known about people's attitudes towards risk‐stratified screening for other cancer types. In Australia, publicly funded national screening programmes exist for breast, cervical and bowel cancer. Opportunistic screening is available for skin cancer^21^ and prostate cancer.^22^

Multifactorial personal risk information is known to increase cancer risk prediction accuracy,^16^ but there is limited research evidence on the acceptability of using such information to guide population screening recommendations. Recent studies suggest that the general public are interested in receiving personal genomic risk information for breast cancer,^11^, ^12^, ^23^ prostate cancer^11^ and kidney cancer^24^ that provision of this information is unlikely to result in high levels of distress and uncertainty^25^ or have a negative impact on cancer worry, anxiety and fear.^26^ Awareness of personal risk factors has also been associated with willingness to participate in bowel cancer screening.^27^

To realize the benefits of risk‐stratified screening, low‐risk individuals (who could still develop cancer) would undergo fewer screenings. This has the aim to balance the goal of cancer detection with the occurrence of harm from more frequent screening,^1^, ^2^ including overdiagnosis^28^ (defined here as cancers that are correctly diagnosed but would not have produced symptoms or been identified clinically^29^). Studies have shown that most people support risk‐stratified screening for breast cancer^11^, ^17^, ^18^, ^19^, ^20^, ^30^ and prostate cancer^11^ more often if required; however, they are reluctant to reduce screening frequency if at lower risk, even when presented with evidence about the potential risks and harms of screening. Varying the frequency of ovarian cancer screening by risk, however, has been shown to be acceptable by the general public.^30^

The screening tests, programmes and the values the public ascribes to screening different types of cancer vary^31^ and could impact on acceptability, as could the experience of receiving personal cancer risk estimates including genomic risk information. Exploration of these issues is ideally suited to qualitative research, to gain an understanding of individuals' perspectives and the reasons for their views^32^. Therefore, this study aimed to explore acceptability of risk‐stratified screening in the Australian population across different cancer types in particular breast, cervical and bowel cancer (public‐funded national screening programmes) and melanoma/skin cancer (a future potential programme), to inform future implementation strategies.

## 2 METHOD

### 2.1 Study design and participants

We conducted a qualitative interview study with members of the public. Participants were purposefully selected to invite for an interview from the database of the Melanoma Genomics *Managing Your Risk Study* ^33^ a two‐arm, parallel‐group randomized controlled trial to evaluate the impact of personal genomic risk of melanoma information on sun‐related prevention and screening behaviours, psychosocial and economic outcomes. Participants were eligible for this qualitative study if they had completed the 12‐month follow‐up in the *Managing Your Risk Study* and given consent to being contacted about future research. Participants in the *Managing Your Risk Study* were initially recruited from the Australian general population through the Medicare database. In the *Managing Your Risk Study,* those in the intervention arm provided a saliva sample, received a booklet with personal genomic risk information derived from a polygenic risk score,^33^ received a call from a genetic counsellor, and a general educational booklet on skin cancer prevention and early detection. Those in the control arm received only the general educational booklet. For the qualitative study, we aimed to recruit participants from the intervention and control arms, to explore potential differences in views in the two groups.

### 2.2 Data collection

Participants in the qualitative study were invited to participate in semi‐structured face‐to‐face or telephone interviews approximately 3‐12 months after completing participation in the *Managing Your Risk Study*. Recruitment was purposive,^34^ with the aim to include a range of characteristics by age, sex, personal genomic risk of melanoma (low, average and high) and geographic location.

Interview guides were developed for control and intervention participants and piloted with three members of the public. Most interview questions were common to both groups (see Appendix 1: Interview guide questions for public participants) and were informed by Proctor et al's conceptual framework of implementation outcomes.^13^ During the interview, participants were asked how they felt about tailored screening for cancer (in the broad sense), in particular to imagine how they would feel about being asked to decrease the frequency of screening or stopping altogether if they were given a low‐risk estimate of developing cancer. This was then asked in relation to different cancer types: breast, bowel, cervical and melanoma/skin cancer. The term ‘risk‐stratified screening’ was replaced by ‘tailored screening’ during interviews to optimize participant understanding of the term.

All interviews were conducted by one member of the research team and lasted between 17 and 49 minutes (mean = 32 minutes). Participants were reimbursed for their time with a $50 AUD gift voucher.

Interviews were first conducted with participants from the intervention group. Two transcripts (third and fourth interviews) were reviewed by three team members and discussed jointly before subsequent interviews. The interviewer met regularly with the research team after completing further interviews to reflect on the process, to review and revise questions to ensure they were being answered in enough depth before remaining interviews were completed. Data saturation was reached when no new information relating to the acceptability of reducing screening frequency for different cancers was identified. Interviews were then conducted with the control group. In discussion with the research team, it became clear that subthemes were similar to the intervention group but reference to specific cancers was limited. Further in‐depth exploration was undertaken through probing questions related to specific cancers in remaining interviews.

### 2.3 Data analysis

Interviews were audio‐recorded and transcribed verbatim. The data were analysed according to thematic analysis as described by Braun and Clarke.^35^ This involved familiarization with the data, generating initial codes using a data‐driven strategy, developing themes by collating codes, generating a thematic map of potential themes and further analysis of the coded data. All members of the research team initially read 4 transcripts (2 intervention and 2 control) to familiarize themselves with the data. Broad top‐level codes were agreed. Analysis was undertaken as one dataset as subthemes were similar. All transcripts across the entire dataset were coded by two researchers and then data were compared across interviews to identify common patterns and initial themes. In consultation with the research team, codes and themes and subthemes were then reviewed and further analysed to summarize the full range of views and experience. This process ensured rigour of analysis as well as coder reflexivity through guidance and discussion. Themes were defined as an important pattern or idea in the data in relation to the research question (ie the acceptability of risk‐stratified cancer screening) and subthemes as a specific idea/s related to the theme.

Files were managed in NVivo 12 (QSR International, Australia) software. The conduct, design and reporting of this study follow the Consolidated Criteria for Reporting Qualitative research (COREQ).^32^

## 3 RESULTS

A total of 40 participants were interviewed (from 80 invited to the interview study); 20 of whom had received personal genomic risk information (10 low risk, 3 average risk, 7 high risk from the intervention group) and 20 who had not (control group). The age range of participants was 21‐68 years and 60% were female. Socio‐Economic Indexes for Areas (SEIFA) scores [for advantage] were above the national average. Table 1 summarizes the participant demographics.

**TABLE 1:** Demographic characteristics of participants

Characteristic	Intervention (N = 20)	Control (N = 20)	Total (N = 40)
Sex			
Female	12	12	24 (60%)
Male	8	8	16 (40%)
Age group			
18‐30 years	4	2	6 (15%)
30‐49 years	8	9	17 (43%)
50‐69 years	8	9	17 (43%)
State			
Victoria	7	5	12 (30%)
New South Wales	5	5	10 (25%)
Western Australia	3	4	7 (18%)
Queensland	3	3	6 (15%)
South Australia	2	1	3 (8%)
Tasmania	0	2	2 (5%)
Risk groups			
Low	10		10 (50%)
Average	3		3 (15%)
High	7		7 (35%)
Socio‐economic index [seifa score]^a^, ^b^			
Mean (sd)	1035.5 (60.6)	1027.8 (70.2)	
Median	1043.6	1031.5	
Min, max	939.2, 1116.9	893.2, 1135.7	

^a^ Area‐based index of relative advantage and disadvantage

^b^ National average SEIFA score = 1000 (standard deviation [SD] 100).

We found that themes were common and views were similar between intervention and control groups. Key themes and subthemes are summarized in Table 2.

**TABLE 2:** Themes and subthemes for acceptability of risk‐stratified screening

Themes	Subthemes
A sense of security	N/A
Tailored screening is common sense	Better health outcomes Timeliness of using personal genomic risk information
Risk and the need to take action	Increasing screening is beneficial Screening for peace of mind Clarity around forgoing screening
Not every cancer is the same	All cancers are important but there is a difference Weighing up inconvenience against the value of screening Personal control over early detection of melanoma and skin cancer
Trust and belief in health messages	Trust in health professionals Scepticism around public health messages Responsibility for health

### 3.1 A sense of security

Overwhelmingly, participants felt that early detection of cancer in the broad sense was important and expressed faith in cancer screening to prevent and detect cancer early. Many described screening as a proactive measure in the ‘battle’ against cancer, often providing a definitive answer.

prevention or recognising and identifying problems early on is obviously far more important than waiting until there are problems. So yeah, I'm very much for prevention or early detection. (Female, 46 years, control group)

For many, screening represented security, removing uncertainty. One participant described having a pap smear combined with the National Cervical Screening Program reminder service as providing freedom from worry.

Yeah, I think it's probably a good safety net and so every time you do screening, you think ‘I'm clear’… So I think it's a good tool and gives you a bit of peace of mind. (Female, 45 years, control group)

### 3.2 Tailored screening is common sense

#### 3.2.1 Better health outcomes

When the concept of tailored cancer screening in the broad sense was explained, almost all participants described tailored screening using personal risk information as a positive and logical progression from the current mostly one‐size‐fits‐all approach of population screening:

…my view is you're going to have better health outcomes when you have personalised management plans or screening plans. (Female, 46 years, low‐risk)

Some felt tailored screening would better motivate individuals to attend screening.

Because some people will think these things won't happen to them, but yeah, if they're more likely to know if they're more at risk, I think it's much more likely to influence people to actually ‐ or motivate them to actually go and get it [screening] done. So I think that's a great idea. (Male, 25 years, low‐risk)

#### 3.2.2 Timeliness of using personal genomic risk information

Many participants thought that using personal genomic risk information to inform their screening risk was timely. Some expressed the importance of embracing new technology to realize the potential benefits, further reinforcing support for tailored screening as a perceived means of improving accuracy and benefiting health.

Awesome idea. I actually think, well it's 2019 people, you know, we've got to get with the program (Female, 56 years, control group)

However, most participants mentioned the importance of confidentiality and privacy despite being happy for this information to determine their risk.

…the individual getting that information I'm okay with, it's when it starts going into a big bucket‐o‐data that I get concerned because once again, it's about the controls around that. (Male, 42 years, average‐risk)

### 3.3 Risk and the need to take action

#### 3.3.1 Increasing screening is beneficial

All participants were in favour of increasing screening frequency if they were found to have an increased risk of cancer. Screening was described as a ‘good thing’ and increasing frequency only added to its value. To one participant, it seemed irrational not to increase screening frequency. When asked if he would screen more often if at higher risk, he responded:

I think I'd probably be stupid not to. (Male, 27 years, control group)

Some highlighted how highly they valued screening, preferring it over other risk prevention strategies. One participant explained that he would be reluctant to give up things he liked such as bike riding and surfing even if at high risk of melanoma but would increase screening frequency.

I'd probably screen more often rather than change my behaviour (Male, 49 years, high‐risk)

A small number of participants did express concern about the potential for unnecessary anxiety as a result of attending screening too often for more than one cancer. One participant discussed this is in relation to two cancers and the potential emotional and logistical burden. She described both inconvenience and worry associated with frequent colonoscopies and the relief when a decrease/change in screening (because of her age) was recommended. As a previous breast cancer patient, she expressed the high level of worry when her on‐going screening for breast cancer was extended from 5 to seven years. She summarized.

If you're liable to be high‐risk of a couple of things, it could be quite concerning to be going for these different tests often, just because you're at high‐risk. (Female, 61 years, control group)

The potential for anxiety related to false‐positives results was also mentioned. In reference to having considered not going as often for mammography screening and cervical screening, one participant explained as follows:

Because sometimes you read about a lot of false positives when you don't actually have breast cancer and probably the mental anguish that you would encounter getting a false positive may not make it worthwhile (Female, 55 years, control group)

#### 3.3.2 Screening for peace of mind

Many participants took time to consider how they felt about reducing screening frequency or not attend screening at all, if they were given a low cancer risk. Most felt it would be acceptable to screen less often but were determined that they would not stop altogether. This was based on low‐risk not equating to no risk and a belief that screening could only have a positive effect. Screening was perceived by some to be part of their responsibility for health.

I think everybody needs screening though, at some point in your life, surely. Like if there's a way to screen for something, even if your risk, is only one per cent, you're still at risk. (Female, 33 years, control)

For some, stopping screening would make them feel unsafe.

…you're automatically going to have an emotional somebody's‐taking‐away‐a‐security‐blanket type of reaction. (Male, 57 years, control group)

Throughout the interviews, participants repeatedly expressed the importance of having the opportunity to be screened at some point.

It would make you feel a bit uneasy and unsure. But obviously it makes sense to have less screening, as long as it kept happening (Male, 21 years, high‐risk)

Being offered a single screening episode as opposed to no screen was seen by some to be a more acceptable option.

Well probably I'd feel better if I at least had one screen, then I might be more happy to go along with it, I'd say (Male, 46 years, control group)

#### 3.3.3 Clarity around forgoing screening

A small number of participants from both intervention and control groups were willing to stop screening (hypothetically speaking), if at low risk and if advised to do so. For these participants, expert and health professional advice around screening, and weighing up potential harms and benefits themselves, would influence their decision to stop or forgo screening. Some felt they were well equipped to make an informed decision about how often to attend screening based on their risk. But these participants felt that others may not be as well equipped, as this participant who had a long history of screening explained:

So I think I would vary the screening based on the risk….That's a very ‐ a reasonably well‐informed decision that I'm making and not everyone else has the level of information or education to be able to do that. (Male, 49 years, high‐risk)

The cost of screening in a public health setting was raised as another reason to forgo screening if at low risk and if advised.

Yes, because if I've got a low‐risk, I don't believe in wasting resources. I believe those resources are important for the people that need them, so if the risk is high, the need is high. If the risk is medium, the testing should be medium, versus low (Female, 67 years, average‐risk)

Participants also felt that having a low risk may diminish worry about cancer and therefore screening would not be a priority.

I probably wouldn't go at all, well I don't need to worry about that, so I wouldn't. Like I mean everyone's got things going on in their lives, that if that's something that's not really a problem or something that they don't have to worry about, well then they wouldn't bother, if they didn't have to (Female, 30 years, low‐risk)

### 3.4 Not every cancer is the same

#### 3.4.1 All cancers are important but there is a difference

When first asked if the type of cancer would make a difference to how they felt about tailored screening, participants were emphatic that cancers were all equally important and therefore given equal attention.

I believe all different types of cancers should be under that sort of same umbrella (Female, 26 years, low‐risk)

It shouldn't be different, a cancer's a cancer (Female, 67 years, low‐risk)

However, screening for breast cancer screening was discussed, particularly by women, with familiarity and respect. Most participants mentioned breast cancer in some way, primarily around genetic risk factors, personal family history and the need for breast cancer screening, reinforcing a high level of awareness in Australia.

I know that there is so much more research for breast cancer and stuff and the screening process is quite refined. (Female, 25 years, low‐risk)

#### 3.4.2 Weighing up inconvenience against the value of screening

When asked if the type of screening test would make a difference to their decision to stop screening if at low‐risk, participants initially thought not. However, when prompted to think about different cancers including breast, bowel, cervical and melanoma, many reconsidered and some speculated that the nature of the test would impact on this decision.

In particular, colonoscopy and Pap smears were reported as good examples of invasive and unpleasant tests that participants would be more willing to give up.

Definitely, if it was something like a colonoscopy that requires a lot of preparation beforehand and you're being sedated and that sort of thing, that would definitely affect ‐ I would not be likely to choose to have something like that regularly. (Female, 33 years, control group)

I mean when they told me I don't have to go and have a Pap smear for five years now, I was like, “great, that's awesome, that's one less thing that I have to worry about”. (Female, 49 years, control group)

However, some just felt the value of the screening would override the inconvenience.

No, I mean as a woman, obviously a Pap smear and a mammogram is more invasive than a skin check or whatever, but that's just what you do. I mean I suppose men have prostate tests and that's probably uncomfortable as well. But I just think… being uncomfortable shouldn't affect you having a test or not. (Female, 49 years, control group)

#### 3.4.3 Personal control over early detection of melanoma and skin cancer

Many participants considered melanoma and other forms of skin cancer to be distinct because they are visible, and therefore, it might be acceptable to stop screening altogether for those at low‐risk.

I guess that's probably one of the benefits of melanomas, is that they're a bit more easily identifiable if you kind of have a rough idea of what to keep a lookout for. But I wouldn't feel the same way about other kind of cancers that aren't visible from the outside. (Male, 27 years, control group)

Many expressed a level of personal control with detecting melanoma early.

I think it's very easy, even if I did get it, I think it would be very easy for me to pick it up pretty quickly, but that's skin cancer, that's something that you can see. (Female, 26 years, low‐risk)

However, participants showed reluctance to give up skin cancer screening if they were already engaged in it regularly and expressed less confidence in their own skills to detect and prevent skin cancer.

I would probably notice that, if it [a mole] was within my sightline, but yeah, I'm not terribly confident in my ability, that's why I make sure I have regular skin checks'. (Female, 33 years, control group)

### 3.5 Trust and belief in health messages

#### 3.5.1 Trust in health professionals

Participants felt that trust in health professionals would impact whether people believe advice about risk‐stratified screening.

I feel like most people would be accepting of that. I think that there's a very high level of trust in health professionals anyway. (Male, 57 years, control group)

And some value was placed on existing relationships with health professionals:

I think if there's a relationship [with a health professional], they're probably more likely to trust it. (Female, 53 years, low‐risk)

However, some participants thought attitudes might be changing as there is now more access to health information.

Trust [in health professionals] has changed… because of Doctor Google, perhaps you might have some reservations, whereas I think probably in the past you would absolutely trust what your doctor said because you didn't have any other information. (Female, 33 years, control group)

#### 3.5.2 Scepticism around public health messages

When asked how they felt about a public health message that recommends reducing or stopping cancer screening for those at low risk, many responded that it seemed illogical and unbelievable.

I don't even think I personally believe in that statement, so I think people would be quite sceptical. I don't think people would want to take that on, or if they hear that, they might say, why would you say that kind of thing. (Female, 25 years, low‐risk)

A participant who had experienced receiving personal genomic risk information, described how risk information would be better provided by a health professional than as a public message.

So I would probably need personalised information as opposed to more general public health information. Then I'd be totally comfortable not doing it. (Male, 49 years, high‐risk)

#### 3.5.3 Responsibility for health

Several participants felt that engaging in screening was part of taking responsibility for one's own health.

…everyone needs to be vigilant to protect themselves and it always comes back on yourself, to how you see yourself and what happens. The buck stops with you, as the old saying goes. (Female, 68 years, control group)

Finally, some were concerned that many individuals do not take enough responsibility for their own health and that reducing screening frequency might reinforce this.

I think that there's an element of risk when you tell someone that they don't need to in that it can make them complacent, or it can make them, I guess feel a lack of sense of responsibility to look after themselves. (Female, 34 years, control group)

## 4 DISCUSSION

These qualitative findings demonstrate that Australian public participants are mostly supportive of risk‐stratified screening across different cancer types, at least insofar as it would lead to additional screening for those deemed to be at high risk. Tailoring screening according to individual risk was described as a logical move to improve screening precision and there was optimism around using personal genomic risk information for estimating risk for this purpose. Consistent with other studies,^11^, ^17^, ^18^ there was strong support for increased screening for those at high risk but not all participants felt that less frequent screening for those at low risk would be acceptable. There were some differences by cancer type and screening test in the level of acceptability of stopping screening altogether for people at low risk.

Our study has some important implications for the implementation of risk‐stratified screening for different cancers. First, some participants were more amenable to reducing screening frequency when the screening test was invasive and uncomfortable. When prompted, participants identified the potential harms of screening tests including anxiety and inconvenience associated with false positives, and the high level of discomfort in some tests such as colonoscopies and pap smears, as reported elsewhere.^20^ However, a significant proportion of the public already opt for unnecessary colonoscopies for bowel cancer screening, a screening intervention that is recommended only for people at increased risk.^36^ A trial is currently exploring the use of a risk‐stratified screening tool to increase risk‐appropriate bowel cancer screening.^37^

Second, offering a single screening episode which could include education about prevention and early detection, as opposed to no screen may be more acceptable to those at low risk, providing a connection to a screening programme. Reluctance to reduce or forgo screening for low‐risk groups was linked to uncertainty and insecurity, given that residual risk exists even for those with a low‐risk estimate; this has also been observed by others.^18^ Most previous research on this topic has been conducted in breast cancer.^12^, ^17^, ^20^ Lippey *et al* found women's reluctance was related to potential loss of reassurance and an emotional connection to breast cancer screening.^20^ A number of other studies note that introducing new cancer screening programmes may receive less opposition than adapting existing ones where the public have an established attachment.^4^, ^20^, ^30^, ^38^ The high level of awareness socially reinforced by high profile campaigns and media coverage of celebrities with breast cancer,^39^ and uncritical support of breast cancer screening observed in our study suggests that it may be particularly challenging to reduce breast cancer screening frequency in countries with established breast cancer screening programmes.

Third, risk‐stratified screening for melanoma (and other skin cancers) may be viewed differently from other cancers, because of its visible nature. Early detection of melanoma in Australia is based on heightened public awareness and opportunistic skin checks but there is currently no formal screening programme.^40^ Risk‐stratified screening for melanoma was acceptable to most, but personal control (self‐efficacy) over its early detection and prevention was seen as an important determinant of whether people would be willing to decrease or stop screening if advised. Participants who already received regular skin checks expressed reluctance to forgo screening even if at low risk.

Concern was also reported about the health of those who opt out of screening altogether.^20^ Some participants in our study expressed concern for and were critical of individuals who do not act to maintain their own health and saw less screening as likely to have negative consequences for this group. This view that holds individuals responsible for their own health is debated ^41^ and may reflect the particular characteristics of participants in this research study who primarily live in areas of socio‐economic advantage.

Other studies have reported a reluctance to forgo screening linked to scepticism that reducing screening frequency was cost‐cutting rather than evidence‐based and in the public's interest.^38^ This was observed in Australia when the National Cervical Screening Program changed from Pap smear tests to human papillomavirus (HPV) testing in 2017 and shifted to a later starting age and larger screening interval.^42^ Audience segmentation, which tailors messages for different groups, is recommended for social marketing campaigns in cancer prevention in addition to whole‐of‐population approaches.^43^ Our findings help to identify these distinct audiences and concerns.

Health professionals are well placed to provide clarity around public messages particularly for low‐risk groups, as participants generally expressed a high level of trust in health professionals. However, trust in health professionals' knowledge about tailored screening is not universal ^23^ and a need to educate health‐care professionals on all aspects of tailored screening has been reported.^44^ Individual expertise and personal experiences impact on risk perception and understanding, and presentation of risks in multiple ways is recommended for effective risk communication.^25^, ^45^ In the Managing Your Risk study, participants were given their remaining lifetime (absolute) risk as both a percentage and a frequency (x out of 100), comparing their personal risk with a person of the same age and gender. It was displayed as icon arrays (100 person diagram) and as a risk category of low, average or high genomic risk. We previously showed that people who received a high genomic risk result for melanoma reported higher distress and uncertainty compared with average and low‐risk groups although overall these levels were low.^25^ Few participants with high‐risk results expressed concerns about life insurance or cancer worry, but participants across all groups frequently mentioned the importance of confidentiality and privacy. The feasibility of providing personal risk information as part of tailored screening through health‐care professionals, such as a triaged approach of general practitioner and genetic counsellor contact according to the level of risk,^46^ also requires further research.

A strength of this study was that it explored people's perceptions about risk‐stratified screening for different cancers, whereas most previous studies have focussed on one cancer type. Our study also provided a rare opportunity to include the views of both those who have experienced receiving personal genomic cancer risk information and those who have not, although they had only received this information for melanoma and not the other cancers. Other than melanoma, we also did not specifically record participants' lived experiences of the cancers being discussed. Another limitation influencing the generalizability of our findings is the potential for bias due to the 50% response rate in this interview study, as individuals supportive of screening may have been more inclined to participate. Indeed, the overall higher socio‐economic status of participants compared with the general population may result in a stronger interest in, and awareness of, cancer prevention and screening than the broader community. Those contacted to take part had previously participated in the parent Managing Your Risk study and nominated their interest in further research, so the response rate may also be related to practical barriers such as responding to a mailed invitation, time or research fatigue. When comparing characteristics among interview respondents and non‐respondents, there were no large or significant differences between them in relation to key demographic or behavioural characteristics.

## 5 CONCLUSIONS

Acceptability of risk‐stratified screening for different cancers may be impacted by the type of screening test, the level of attachment to the screening programme and the individual cancer itself, all of which have implications for implementation. Findings from this study indicate that acceptance of risk‐stratified screening is high, but most people are unlikely to want to forgo screening altogether even if at low‐risk. Approaching individual cancer types differently, tailoring messages for different audiences, fostering public trust in screening programmes and understanding the public's reasons for participating in screening may assist with designing future implementation strategies for precision screening.

## CONFLICT OF INTEREST

The authors declare that they have no conflict of interest.

## AUTHOR'S CONTRIBUTIONS

KD, AEC, NMR, LK, AKS and AJN were involved in the concept and design of the study, development of methodology, participated in data analysis and interpretation and revised the manuscript critically. In addition, KD (PhD candidate; experienced health educator) conducted the interviews (40) and wrote the manuscript. ZS was closely involved in data analysis and manuscript revision. AEC, NMR, LK and AJN provided study supervision. All authors read and approved the final manuscript.

## ETHICAL APPROVAL

Ethics approval was obtained by University of Sydney Human Research Ethics Committee (HREC) 2018/941.

## Data Availability

The data that support the findings of this study are available on reasonable request from the corresponding author. The data are not publicly available due to privacy or ethical restrictions.

## APPENDIX 1 Interview guide questions for public participants [intervention and control groups]

**Table:**

*Recently you participated in a study about managing your risk of melanoma*. What was your experience of the study? Can you tell me about any cancer screening you have had? How did you feel about it? *We are starting to move towards a more tailored personalised approach to screening. For example, at the moment everyone over 50 gets sent a bowel kit every two years to test for early signs of cancer. One‐size‐fits‐all. Bowel cancer screening is tailored in some ways because those with a strong family history start screening earlier and might have a colonoscopy. If more risk factors were included (more than age and family history) the idea is that advice could then be more precise –**tailored to the individual*. What do you think about this idea of personalised screening‐ tailored to the individual? How do you feel about different risk factors being used to inform your personal screening? *(by risk factors we mean things that increase the chance of developing cancer such as lifestyle, environment and information within your genes)* How do you feel talking about your cancer risk and the different factors? How do you feel about your personal genetic (genomic) risk information being used to inform your personal screening advice? If you were given a higher chance of developing cancer, would you agree to undergo a screening test more often? If you were given a low chance, would you agree to undergo screening less often or perhaps not all? How would you feel about that? Do you think you feel differently about this for different cancers? How do feel about screening less often for breast cancer; bowel cancer; cervical cancer and skin cancer? How do you think the type of screening test would affect this? How do you feel about reducing how often you screen in a formal screening program, where we already have screening advice? [breast cancer, bowel cancer, cervical cancer?] What about for melanoma? How well do you think people would trust a health recommendation that less screening is beneficial overall? If your personalised screening program recommended lifestyle changes, how would you feel about making those changes?
